# Unraveling the Layers: Dissociative Identity Disorder as a Response to Trauma

**DOI:** 10.7759/cureus.60676

**Published:** 2024-05-20

**Authors:** Karlyle Bistas, Ramneet Grewal

**Affiliations:** 1 Behavioral Health, Wayne State University, Detroit Medical Center, Detroit, USA; 2 Medicine, Saba University School of Medicine, The Bottom, NLD

**Keywords:** split personality disorder, multiple personalities, dissociation, psychological responses, dissociative disorders

## Abstract

Dissociative identity disorder (DID), previously recognized as multiple personality disorder, impacts approximately 1.5% of the population. The Diagnostic and Statistical Manual of Mental Disorders, Fifth Edition, Text Revision (DSM-5-TR), outlines various dissociative disorders (DDs), including depersonalization/derealization disorder, dissociative amnesia, DID, unspecified DD, and other specified DDs. Among these, DID stands out as the most severe, characterized by persistent depersonalization/derealization and dissociative amnesia. This case report explores the symptomatology of DID, available treatments, and the role of trauma.

## Introduction

Dissociative disorders (DDs) are more prevalent than some of the commonly assessed psychiatric disorders [1]. Dissociative identity disorder (DID), previously referred to as multiple personality disorder until 1994, affects approximately 1.5% of the global population [2]. The Diagnostic and Statistical Manual of Mental Disorders, Fifth Edition, Text Revision (DSM-5-TR), categorizes several DDs, including depersonalization/derealization disorder, dissociative amnesia, DID, unspecified DD, and other specified DDs [1]. DID stands out as the most severe form of DD, characterized by persistent depersonalization/derealization and dissociative amnesia [1]. In the DSM-5-TR, DID is described as a disruption of identity featuring two or more distinct personality states or an experience of possession [3]. DID is an uncommon condition associated with severe symptomology. Individuals with DID often face multiple crises, frequently involving self-injurious behavior and substance use [4]. DDs typically disrupt normal consciousness, memory, identity, and behavior [4]. DDs can be classified into positive and negative symptoms, with positive symptoms involving phenomena such as new personalities and derealization [4]. In contrast, negative symptoms include superficial displays of emotion and a lack of motivation [4].

DID is a subset of the broader DDs spectrum, delineated by specific criteria outlined in the DSM-5-TR [4]. The presence of two or more distinct personality identities serves as a hallmark of DID. Each persona may have a unique name, history, and personality traits. For DID to be recognized, at least two identities must be discerned. Each identity displays consistent patterns in perceiving the world, forming relationships, and interpreting surroundings [5]. Frequently, one identity lacks awareness of the events that occurred while another is in control [6]. Several etiological factors likely contribute to the development of DID, including abuse, emotional neglect, disrupted attachment, and boundary violations. The trauma leading to DID can stem from familial, societal, and cultural sources, shaping both its occurrence and expression [7]. Cultural processes also play a significant role in shaping the development and phenomenology of DID [4]. Understanding the influence of culture involves two main aspects: its role as a source of trauma and its impact on how the disorder manifests [4].

The emergence of DID seems to correlate with severe child abuse, disorganized attachment styles, and a lack of social and familial support. The tendency to dissociate appears to be influenced not only by inherent temperament and genetics but also by a dysfunctional family structure and attachment disorder acquired during early childhood [4]. Parenting styles often observed in these cases tend to be authoritarian and rigid, paradoxically featuring an inversion of the typical parent-child relationship [8]. DID is a complex developmental condition arising from post-traumatic experiences. In the DSM-5-TR, the DDs chapter is strategically positioned after the chapter on trauma and stressor-related disorders, acknowledging the link between DDs and psychological trauma [3]. The core features of DID often manifest alongside a mix of psychiatric symptoms, which, rather than dissociative symptoms, are usually the primary concerns of the patient [9]. The diagnosis of DID requires a thorough examination of personal history by both psychiatric professionals and experienced psychologists. Individuals with DID often encounter misdiagnosis, frequently being erroneously categorized with other personality disorders, particularly borderline personality disorder (BPD), because of the prominent presence of dissociative elements and amnesia in BPD. Accurate diagnostic assessments often require longitudinal evaluations over extended periods, involving detailed history-taking, and multiple sources are typically consulted to gather a comprehensive history. This case report explores the treatment of a patient diagnosed with DID in an outpatient setting. It examines both the presentation of DID and the influence of trauma on this diagnosis.

## Case presentation

The patient was a 49-year-old male with a past psychiatric history of alcohol use disorder and psychosis, not otherwise specified, seeks outpatient care for medication management. During a mental status examination, the individual appeared appropriate for their age, with average stature, overweight, and dressed suitably. Their speech was within normal parameters regarding cadence, volume, and tone. The patient is currently prescribed and taking risperidone 0.5 mg nightly for psychotic symptoms and buspirone 10 mg twice daily for anxiety. He was prescribed ziprasidone 20 mg twice daily with food, which he took intermittently over a year, but it was discontinued because of excessive sedation. This medication was chosen because of its more favorable metabolic profile but was switched to risperidone 0.5 mg nightly (he was briefly on this in the past, unknown dose or time). He had not done therapy in the past. It was acknowledged that firearms were accessible but stored safely, and there were no immediate suicidal thoughts present. The individual has undergone substance rehabilitation and reports abstaining from alcohol for several years. Infrequent cannabis use is mentioned.

Their family history includes bipolar disorder and schizophrenia, on the maternal side. Childhood exposure to significant physical trauma is acknowledged. The patient recounts experiencing periods of neglect during which he lacked access to both food and water. Additionally, from his earliest memories through his teenage years, he endured severe physical abuse. The individual describes experiencing 11 distinct personality types with associated traits and names. He describes experiencing 11 distinct personalities, yet he provides detailed accounts of only two. One personality, labeled "Macho Man," emerged when he felt undermined or threatened, exhibiting directness, loudness, and confidence. Another personality he identified was the "Security Guard," activated when he sensed he was being followed or watched, adhering strictly to rules and ensuring compliance. While it remains uncertain if he had 11 distinct personalities, this number was inconsequential as the diagnosis only necessitated two or more distinct personalities. Laboratory findings include elevated hemoglobin A1C at 6.2% (below 5.7% is normal), cholesterol of 240 mg/dL (a value of below 200 mg/dL is normal), and low vitamin D levels of 10 ng/mL (normal is between 20-40 ng/mL). During the assessment, the individual presented calmly and cooperatively and was awake, alert, and oriented to person, place, and time. He denied experiencing visual hallucinations but reported auditory experiences related to their multiple personalities. The patient was unable to elaborate further on the auditory hallucinations he was experiencing. The patient had limited insight into his mental health condition. During sessions, there are observed instances of short-term memory lapses and occasional dissociative episodes, in which the patient would be unresponsive to questioning for a few minutes. In 2023, the outpatient psychiatrist established the diagnosis by thoroughly reviewing and aligning it with the criteria outlined in the DSM-5-TR.

Currently, the patient is seeing their outpatient psychiatrist every month. He is psychiatrically stable but continues to struggle with multiple personalities. He is sleeping better and is less paranoid. He is unwilling to do therapy at this time.

## Discussion

Signs and symptoms of DID 

Symptoms of DID typically involve the presence of two or more distinct personality states, along with frequent memory gaps that hinder the recollection of significant life events or details. According to DSM-5-TR criteria, criterion A outlines this disruption of identity, marked by the presence of multiple distinct personality states, while criterion B highlights the memory gaps in everyday recollections [3]. These symptoms lead to significant distress or impairment and are not attributable to cultural or religious practices, substance use, or other medical conditions. Individuals with DID often experience recurrent episodes of amnesia, identity confusion, and feelings of detachment from their own body (depersonalization) or surroundings (derealization) [3]. They may struggle to recognize themselves and may undergo abrupt mood shifts, as each personality tends to possess its unique emotional characteristics [3]. Additionally, patients may endure flashbacks, intrusive thoughts, self-harming behaviors, or suicidal ideation [3]. The impact of these dissociative episodes often disrupts the individual's ability to function in various domains, such as work, school, and relationships [3]. Co-occurring mood disorders are frequently observed in patients with DID.

Review of current literature 

Lauer et al. proposed a connection between DID and BPD [10]. They noted minimal differences in symptoms between these psychiatric conditions [10]. Instead, they characterized a collection of symptoms observed in individuals with disturbed personalities [10]. Gillig discussed post-traumatic stress disorder (PTSD) symptoms in DID patients, likely stemming from childhood sexual abuse [8]. The "spontaneous age regression" observed in DID was linked to early trauma, with PTSD symptoms playing a central role [8]. Commonalities between BPD and DID suggest a potential link through shared experiences of trauma. Horevitz et al. discovered that 70% of DID-diagnosed patients, upon chart review, could equally meet BPD criteria [11]. Similarly, it was found that 64% of DID patients also fulfilled BPD criteria [10]. Studies propose that DID manifests as a syndrome in individuals grappling with various personalities, suggesting a continuum shared by BPD and DID [10]. The relevance of the socio-cognitive model in DID diagnosis has spurred debate and research [12]. This model, emphasizing the interplay of personal factors, behavior, and environment in shaping behavior, has been subject to scrutiny [12]. Furthermore, certain research indicates that this model fails to recognize DID as a genuine psychiatric disorder [13]. Dalenberg suggests that clinicians might inadvertently validate their patients' alternate personalities, potentially fostering the disorder's development [13]. In 2013, the American Psychiatric Association introduced a dissociative subtype to the PTSD diagnosis, marked by enduring depersonalization and derealization [1].

Pathophysiology and risk factors for DID 

The complete pathophysiology behind the development of DID remains only partly understood. Environmental factors are believed to be linked to DID, with no identified biological causes. Putnam and his colleagues propose that DID arises from traumatized children's inability to form a cohesive sense of self, leading to the emergence of alternate identities [4]. This phenomenon is especially apparent when traumatic experiences occur before the age of five [4]. DID seems to stem from prolonged and intense stress and trauma. Severe childhood abuse, disorganized attachment styles, and lack of familial support during traumatic experiences are often precursors to DID [8]. The inclination towards dissociation seems linked to dysfunctional family structures and attachment disorders in early childhood [8]. Early childhood trauma appears to correlate with disturbances in attachment [1]. Alternate personas emerging as a response to traumatic events become ingrained roles for individuals. DID is commonly linked with significant childhood trauma and abuse [4]. This trauma can manifest in various forms, including physical, emotional, or sexual abuse [4]. Widely regarded by many researchers and psychiatrists as the most severe form of childhood-onset PTSD, DID is perceived as a protective mechanism triggered by trauma. It shares many characteristics with PTSD but encompasses identity disruption in multiple distinct personalities [14].

Two opposing theories exist regarding the relationship between trauma and dissociation: the trauma-related and fantasy-prone models [14]. The trauma-related model underscores trauma's profound impact, positing that dissociative tendencies develop as a coping mechanism to navigate traumatic experiences [14]. Dissociation serves as a survival strategy, shielding the mind from the overwhelming emotional and psychological repercussions of trauma [14]. This defense mechanism involves compartmentalization, emotional numbing, and detachment from traumatic emotions, often leading to amnesia and memory gaps [14]. While dissociation may offer short-term benefits, persistent and severe dissociation can have lasting effects on mental health [14]. Conversely, the fantasy-prone model suggests that dissociation arises from an individual's vivid imagination and inclination toward fantasy [14].

Prevalence of DID 

DDs affect 1%-5% of the global population [14]. In the United States, the 12-month prevalence of PTSD is 4.7%, with 14.4% of PTSD cases meeting the criteria for the dissociative subtype [1]. DID is observed in 1%-1.5% of the general population [4]. Patients often undergo treatment for five to 12.5 years before receiving a DID diagnosis [14].

Evaluation and diagnosis of DID 

Diagnosing any psychiatric disorder requires clinical judgment. Objective scales are available for diagnosing DID. The Dissociative Experiences Scale, a 28-item self-report tool, serves as a screening instrument, evaluating a wide range of dissociative experiences, from problematic to normal, such as daydreaming [14]. The Dissociation Questionnaire (DIS-Q), comprising 63 questions, measures identity confusion and fragmentation [14]. Additionally, the Difficulties in Emotion Regulation Scale (DERS) comprises 36 questions focusing on challenges in impulsivity and emotional responses to situations [14]. Assessing and diagnosing DDs can be challenging because of trauma-related symptoms [1]. Validated clinical interviews and measures aid in differentiating DDs from other psychiatric disorders [1]. Several self-report measures are available for dissociation. 

Some of the questions in The Dissociation Questionnaire are: “Do you ever feel like you're watching yourself from outside your body?” “Have you ever had periods of time where you feel like you're disconnected from your thoughts or feelings?” Some of the questions in the DERS are “When I'm upset, I have difficulty controlling my emotions.” “I have difficulty accepting my feelings, whether they are positive or negative.” Although these questionnaires are helpful tools, they are not used for diagnosis. The patient was diagnosed using the DSM-5-TR criteria, and scales were not used.

Evidenced-based treatment for DID 

Individuals who have access to treatment often experience a significant reduction in symptoms [1]. Treatment for DID typically follows a three-pronged approach: establishing safety, stabilization, and symptom reduction; addressing and integrating traumatic memories; and promoting identity integration and rehabilitation [14]. Prioritizing patient safety, including managing suicidal or homicidal ideation, is paramount in psychiatric interventions. Another key aspect of DID treatment involves processing and integrating traumatic memories [14]. Trauma-focused therapies, such as cognitive behavioral therapy (CBT) and dialectical behavioral therapy (DBT), are crucial [14]. DBT considered the gold standard for BPD has shown efficacy in treating DID because of symptom overlap between the two disorders [14]. Therapy aims to unify the various personalities and enhance patient awareness [14].

Eye movement desensitization and reprocessing (EMDR) also plays a significant role in DID treatment. EMDR aims to alleviate the distress associated with traumatic memories by employing specific eye movements. It can be utilized for symptom reduction, containment, ego strengthening, and addressing alternative identities [14].

Psychopharmacology is not the primary treatment for DID but may be used to alleviate associated symptoms. Various medications, including antipsychotics, mood stabilizers, and stimulants, have been utilized [14].

Neuroanatomy associations 

Neuroimaging studies reveal intriguing structural and functional changes in the brains of individuals with DDs, which are both similar to and distinct from those observed in PTSD. These findings contribute to a deeper understanding of the distinct characteristics and treatment requirements of these individuals. A comprehensive review of research in this domain covers structural MRI, resting fMRI, and task-related fMRI studies across diagnostic groups, including PTSD, BPD, depersonalization/derealization disorder, and DID [1]. In DID, there are aberrations in the temporal and frontal cortices. Patients with DID and dissociative PTSD showed more overlap in brain activation in these areas than DID [1]. Attempts to replicate brain activation patterns using professional actors with varying levels of fantasy-proneness, trained to simulate dissociative symptoms, have failed to reproduce these distinctive patterns [1].

Associations with other mental illnesses

Tezcan et al. discovered that individuals diagnosed with DID frequently exhibit comorbid psychiatric disorders [15]. Common differential diagnoses for DID include BPD, histrionic personality disorder, PTSD, and psychotic disorders such as schizophrenia [14]. BPD and DID commonly manifest symptoms of dissociation and amnesia, with BPD being the most frequent alternative diagnosis [14].

Limitations and future directions

There are limitations to this study report such as the generalizability of the study. DID occurs in 1-5% of the global population, and it is considered a relatively rare psychiatric diagnosis [14]. In rare cases, it may be not easy to represent broader populations. Therefore, findings from case reports may not always be generalizable. This case report does allow the reader to reflect on DID, as it has fallen out of popularity in psychiatric diagnoses. As DID is associated with long-term exposure to trauma, it should be investigated how interventions can be applied earlier to prevent a full-blown diagnosis. This study underscores the need for further research into the neuroanatomical links between DID and trauma as well as potential variations in treatment approaches based on its etiology.

Case discussion 

The patient's history of trauma posed a risk factor for the development of DID. It seems that during childhood, he experienced a profound sense of helplessness, leading his various personalities to adopt the roles of authority and control. Such trauma can come from common experiences such as bullying. Studies have shown instances of DID emerging in individuals who endured bullying. For instance, Zakaria et al. presented a case report depicting a patient who began dissociating as early as age five, triggered by traumatic events following conflicts and bullying in school [16]. This case is important to note, as DID is often thought to be associated with severe trauma.

## Conclusions

This case report delves into the intricacies of DID, a rare condition associated with severe behavioral health symptoms. The authors aim to explore the connections between DID and other mental disorders while also proposing potential treatment approaches. Recognizing whether DID is an independent disorder or symptomatic of another mental health condition is vital for informed diagnostic and treatment decisions. The case study highlights the challenges in diagnosing DDs, emphasizing the lack of training among healthcare professionals in this area. Additionally, it acknowledges the study's limitations and underscores the need for further research into the neuroanatomical links between DID and trauma as well as potential variations in treatment approaches based on its etiology.
